# Emotional intelligence: predictor of employees’ wellbeing, quality of patient care, and psychological empowerment

**DOI:** 10.1186/s40359-021-00593-8

**Published:** 2021-06-04

**Authors:** Leila Karimi, Sandra G. Leggat, Timothy Bartram, Leila Afshari, Sarah Sarkeshik, Tengiz Verulava

**Affiliations:** 1 School of Psychology and Public Health, La Trobe University, Plenty Rd, Bundoora, VIC Australia; 2 School of Management, College of Business, RMIT University, Melbourne, Australia; 3 School of Business, La Trobe University, Melbourne, Australia; 4 School of Medicine and Healthcare Management, Caucasus University, Tbilisi, Georgia

**Keywords:** Emotional intelligence, Wellbeing, Psychological empowerment, Quality of patient care

## Abstract

**Background:**

The study explored the role of emotional intelligence (EI) on employees’ perceived wellbeing and empowerment, as well as their performance, by measuring their quality of care.

**Methods:**

The baseline data for the present project was collected from 78 staff of a Victorian aged care organization in Australia. Self-administered surveys were used to assess participants’ emotional intelligence, general well-being, psychological empowerment, quality of care, and demographic characteristics. The model fit was assessed using structural equation modelling by AMOS (v 24) software.

**Results:**

The evaluated model confirmed that emotional intelligence predicts the employees’ psychological empowerment, wellbeing, and quality of care in a statistically significant way.

**Conclusions:**

The current research indicates that employees with higher EI will more likely deliver a better quality of patient care. Present research extends the current knowledge of the psychological empowerment and wellbeing of employees with a particular focus on emotional intelligence as an antecedent in an under-investigated setting like aged care setting in Australia.

## Background

Today's organisations are under increasing pressure to expand the quality of work and ability to compete in the workplace that is continuously changing. These changes involve increased dependency on social skills and new technologies, continuous competency development, risk-taking, networking, and innovation. They also include changes in organisational structure and relationships, such as reduced hierarchies, blurred boundaries, moves to teams as basic building blocks, and increased complexity of work. As the current pandemic has shown, they also embrace profound and fast changes to the way we work in the face of crises and organisations are looking at new strategies to promote such qualities as wellbeing, psychological empowerment and work engagement which are antecedents of job satisfaction and quality of patient care [1–3].

There is strong evidence that EI is an important factor in improving work performance [4]. Research indicates that higher EI leads to enhanced psychological wellbeing and higher rates of positive emotional states [5–7], and that emotional intelligence training can develop meaningfulness at work and happiness [8, 9]. In a meta-analysis, O'Boyle et al. [10] found overall validity for three streams of EI research (ability measures, self- and peer-report measures, and mixed models) predicting job performance equally well. EI also influences the success with which employees interact with colleagues, the strategies they use to manage conflict and stress, and positively contributes to several aspects of workplace performance [11].

Researching the relationship between EI and job satisfaction among nurses, Gong et al. [12] examined the mediating effect of psychological empowerment and work engagement in this association. Using structural equation modelling, they found that high trait EI may improve occupational wellbeing through the chain-mediating effects of these two constructs. A 2017 meta-analysis of EI and work attitudes has found that all three types of EI are significantly related to job satisfaction [13]. The results indicate that workers with higher EI have higher job satisfaction, higher organizational commitment, and are less likely to change jobs. Another recent study has found statistically significant positive relationships between EI, empowering leadership, psychological empowerment and work engagement [14]. This finding suggests that EI training of health workers to improve psychological empowerment and work engagement could help their organisations to improve their relationships with patients, provide better care, and reduce staff turnover. Emotional intelligence may be most important in the service sector and in other jobs where employees interact with customers. Several studies found a positive association between the EI of nurses and service quality and patients' compliance with care [15–17].

There is evidence that communication effectiveness and job satisfaction of the employees are related to their managers' EI [18]. Research shows that leaders who build effective interpersonal relationships with those in lower rank are using EI to lead individuals to work more effectively and with increased overall job satisfaction [19, 20]. Udod et al. [21] found that leaders who use EI to build interpersonal relationships with their subordinates achieve higher overall job satisfaction and better work effectiveness among those employees. These positive changes are strongly influenced by the leaders who value and respect their employee’s opinions, abilities, personal emotions, and character. Increased empowerment was directly related to the support and level of autonomy given by the leader and a work environment allowing career growth and development [21].

There has been much interest in empowerment in the workplace for a variety of reasons. Studies found that empowering subordinates contributes to managerial and organisational effectiveness. There is a significant relationship between psychological empowerment and work engagement. Alotaibi et al. [14] investigated the role of EI and empowering leadership (EL) in improving psychological empowerment and work engagement. They found significant positive relationships between EI, EL, psychological empowerment and work engagement, suggesting that EI is a good predictor of EL and psychological empowerment, while EL supports work engagement.

Staff empowerment is linked to work behaviours, attitudes, and performance. It tends to have a direct effect on performance and indirect effects through its influence on job satisfaction and innovativeness [22]. In healthcare, employee empowerment denotes the level to which caregivers have the authority to make decisions, such as evaluating the patient condition and determining the most suitable treatment. A review of studies exploring the effect of structural empowerment of nurses on quality outcomes in hospitals found that there are positive associations between the structural empowerment of nurses and the quality of outcomes, such as patient safety, work effectiveness, efficiency, and patient‐centeredness of patient care in hospitals [23].

Quality of healthcare can be defined in many ways. The Institute of Medicine defines quality as the "degree to which health services for individuals and populations increase the likelihood of desired health outcomes and are consistent with current professional knowledge” [24]. A more recent study defined quality of healthcare, using various healthcare stakeholder perceptions and expectations, as “consistently delighting the patient by providing efficacious, effective and efficient healthcare services according to the latest clinical guidelines and standards, which meet the patient's needs and satisfies providers” [25].

Many nursing studies have shown an association between EI and nurses' quality of care. A 2017 study examining the relationship between patient satisfaction and EI skills of nurses found a strong correlation between the satisfaction scores and emphatic concern, utilization of emotions, and emotional awareness subheadings of the patients [26]. A 2018 study, exploring the role of EI in engagement in nurses, found that nurses with higher levels of EI also scored higher in engagement. The greatest predictor of engagement was the interpersonal factor [27]. A study investigating emotional labour among Australian community nurses found that emotional labour and EI predicts wellbeing as well as job-stress [28]. With the current shortage in the nursing workforce, effective EI training may provide the key to keeping nurses in their jobs while helping them reduce job-stress and burnout levels. Emotional intelligence also seems to correlate highly with wellbeing in nurses, has a positive correlation with work performance and the ability to positively affect patient safety [29–31]. Today, EI is one of the most sought-after skills in the workplace. When it comes to healthcare workers and nurses, increased EI may save lives, not to mention relieve stress.

The model fit was in this study was assessed using structural equation modelling (SEM). SEM has been used successfully in research involving EI and nurses. For example, a 2016 study used SEM to analyse the goodness of fit of the hypothetical model of nurses' turnover intention. The results suggest that increasing EI in nurses might significantly decrease nurses' turnover intention by reducing the effect of emotional labour on burnout [32]. Another study used SEM to examine the mediatory role of positive and negative affect at work. The researchers found that these mediate the relationship between EI and job satisfaction with positive affect exerting a stronger influence [33].

The present research project investigated the importance of EI as an antecedent to wellbeing, psychological empowerment and quality of care. The research is one of the few studies in Australia in a much under-researched area of aged care setting. It contributes to international literature by examining the EI link with the three constructs. Thus, it was hypothesised that:

- Higher emotional intelligence is a predictor of better wellbeing,
- Higher emotional intelligence is a predictor of psychological empowerment,
- Higher emotional intelligence leads to better quality of patient care among aged care staff.

## Methods

This study aimed to further explore the role of EI on psychological empowerment and the quality of care and wellbeing in an aged care setting. The current research used a sample of 78 participants of a Victorian aged care facility. The workers from all levels of the organisation having contact with the residents were included, including personal care workers (PCW), nurses, and lifestyle, food and safety staff.

The demographic characteristic of the staff are detailed in Table 1. The staffs' age on average was 45.7 years, and they had almost 12 years of experience of working in their position, with 25 working hours per week. Majority of the staff were female (82%), nursing and personal care workers (61%).

**Table 1:** Characteristic of the participants

	N	Mean	Std. deviation
Age of participants	73	45.6	11.5
Years of job experience	77	12.3	10.5
Hours worked per fortnight	74	50.7	18.2

	n	Percent	
Position			
PCW	26	34.2	
Nurse	22	28.9	
Food/domestic services	17	22.4	
Leisure/lifestyle	5	6.6	
Management, physios and others	6	7.9	
Sex			
Male	14	18.0	
Female	63	82.0	

### General wellbeing

The *General Well-being Questionnaire* (GWBQ), developed by Cox et al. [34]. The GWBQ is a scale with 24 questions that assess general malaise frequency on a 5-Likert response where a high score is indicating lower wellbeing.

### Psychological empowerment

*Psychological empowerment Scale* [35] for evaluating the perceived empowerment on a 5-likert response using 12 statements, where higher score represent higher level of empowerment.

### Patient satisfaction

Patient Satisfaction with Nursing Care Quality Questionnaire (PSNCQQ) [36] was used to measure the quality of patient care. The terminology was modified slightly to make it suitable for use in an aged care population. Seventeen items were adapted (two items related to the discharge, and after-discharge coordination were removed as were not relevant to the aged care setting); higher scores refer to a higher quality of patient care.

### Emotional quotient inventory (EQ-i 2.0®)

The *EQ-i 2.0* used in this study which assesses the social and emotional elements [37]. Sing 133 questions on a Likert response of 1 (never/rarely) to 5 (always/almost always). The EQ-i 2.0 is a self-report measure to measure constructs related to EI. A total score of the EQ-I 2.0 was used in this study to measure emotional intelligence (EI).

### Validity and reliability

All the surveys used in this study are pre-validated scales. However, the reliability of the scales was also assessed in this study. The study scales showed excellent reliability: The General Wellbeing Questionnaire (GWBQ) (α = 0.92) and Psychological Empowerment (α = 0.92), PSNCQQ (α = 0.91).

### Ethical considerations

The Human Research Ethics Committee of the participating organisation was obtained for this study.

### Data analysis

Structural equation modelling (SEM) by AMOS (v 24) software was used to assess the model fit. Chi-square as a goodness of fit statistics provides a good description of the data. A non-significant chi-square means the proposed hypothesis of model fit is supported, and the null model (no relationship between constructs) is rejected. However, chi-square is highly influenced by sample size; therefore, a more robust measure of the relative chi-square (CMIN/DF) is used for model fit evaluation. Besides, other fit indices are proposed, including the root-mean-square error of approximation (RMSEA), comparative fit index (CFI) and the Akaike Information Criterion (AIC). RMSEA is the suggested fit indices representing absolute fit; CFI is recommended for model comparison. A combination of these fit indices, such as CMIN/DF, RMSEA and CFI, are commonly used in research. The AIC is another measure of fit that was used for this study. The smaller value of AIC indicates a superior model fit [38].

### Normality and bootstrapping approach

The normal distribution of data and outliers were assessed before proceeding with the model fit evaluation. Although the data were within the normal threshold at univariate level (where kurtosis and skewness were smaller than 7 and 3 in order), multivariate critical ratio and kurtosis were greater than 1.96 and 5 in order, indicating violation of the normality assumptions. Hence the bootstrapping procedure was implemented due to violation of normality and relatively small sample size (n = 78), to assess the parameter estimates. The number of bootstrapping subsamples needs to be high enough to deliver valid results. They must be higher than the number of valid observations in the original dataset (in this study, higher than 78). As a general rule, 500 bootstrap samples are recommended in SEM [39]. In this study, the bootstrapping procedure and the Bollen-Stine bootstrap procedure were implemented to test the proposed model.

## Results

### Model evaluation

**Fig. 1:**
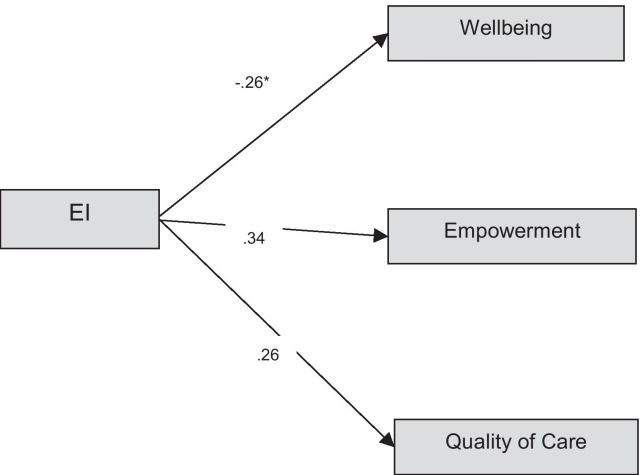
Path coefficients of the proposed model. *Note*: *higher score in wellbeing represents lower wellbeing and high illness

Figure 1 presents the model used in this study and evaluated by SEM. The model fit evaluation indicated χ^2^/df less than 3 which represents great goodness of model fit (χ^2^/df (19) = 1.39). The fit indices in Table 2 also show acceptable fit for the model (CFI = 0.96, RMSEA = 0.07 (0.05, 0.06). RMSEA less than 0.08 and 0.05 & CFI of greater than 0.90 and 0.95 were considered as marginal and good fit respectively for this study. AIC for the proposed model was significantly smaller which indicated a better model fit (AIC = 76.53). The standardised regression weights were all significant (p < 0.05) and presented in Table 3.

**Table 2:** Summary of Fit Indices

Model	χ^2^	df	χ^2^/df	AIC	CFI	RMSEA
The proposed model	26.53	19	1.39	76.53	0.96	0.072
Independent model	233.18	28	8.32	265.18		

^*^ χ^2^/df less than 3 represents acceptable fit; RMSEA less than .08 and .05 & CFI of greater than 0.90 and 0.95 were considered as marginal and good fit respectively

The paths of factor loadings of emotional intelligence to wellbeing, quality of care and psychological empowerment were all significant (p < 0.05). Figure 1 presents the standardised factor loadings of the model. Psychological empowerment deemed to be significantly related to EI skills (β = 0.34). Quality of care was correlated positively with EI skills where higher EI skills were associated with a higher level of quality of care (β = 0.26). Wellbeing (ill health) was negatively associated with EI skills where higher EI associated with the lower level of illness (β = 0.26).

The bootstrapping procedure showed relatively stable parameter estimates, demonstrating the validity of the results. The Bollen-Stine approach showed that the evaluated model was not significantly changed from the model resulting from 500 bootstrapping samples (p = 0.13).

## Discussion

The present project, aimed to explore the role of EI with wellbeing, quality of patient care and psychological empowerment among a group of Australian aged care employees. The evaluated model confirmed that emotional intelligence is related to all three variables in a statistically significant but moderate way. Both psychological empowerment and quality of care were significantly related to EI skills. Wellbeing (ill health) was significantly predicted by EI skills.

This study shows that those with a high level of EI are possibly more psychologically empowered.

According to Spreitzer [35] and Kanter [40], psychologically empowered employees are driven by intrinsic motivation, and they are more likely to perform effectively [22, 41]. However, the results need to be treated with caution because SEM-based analyses reported here are estimates based on cross-sectional data; they do not provide sufficient evidence to demonstrate the existence of a causal relationship.

The results also suggest that if EI is related to employees' wellbeing, empowerment and quality of care, then implementation of interventions for employees in the healthcare sector to learn and practice EI skills seem to be valuable for employee empowerment and consequently for enhancing employees' performance. This finding has been substantiated by an integrative literature review by Kline et al. [42] who found that EI is central to nursing practice and should be included in nursing education. Evidence shows that EI impacts on ethical decision-making, critical thinking, evidence and knowledge use in practice.

The study also provided evidence for a significant association between EI and wellbeing of employees, demonstrating that staff with higher EI are more likely to have better emotional and psychological wellness. This finding is in line with recent studies such as Karimi et al. [28], [43], that reported a significant relationship between EI and wellbeing among nurses and aged care staff.

Finally, the findings indicate that the employees' EI is related to the quality of the care for the aged care residents. Specifically, the care provided by emotionally intelligent employees is more likely to result in desired outcomes for the residents and consequently in an increased level of residents' satisfaction with the quality of patient care.

Our study extends the current knowledge of psychological empowerment and wellbeing of employees with a particular focus on emotional intelligence as an antecedent. Our findings demonstrate that employees' emotional intelligence not only relates to the residents' satisfaction with the quality of patient care but also seems to be associated with employees' psychological empowerment and wellbeing. This indicates that EI is an important skill to be learnt in order to generate the desired outcomes for two main stakeholders in the aged care sector: the residents and the employees.

### Limitation

Although bootstrapping procedure used to report more stable and valid results, the relatively small sample size and lack of previous studies on Australian aged care made the generalizability of the findings limited. Future studies on aged care setting with a bigger sample size is recommended. In addition, longitudinal studies assessing the EI training skills on employees' mental health and performance is strongly recommended in order to shed more light in this area.

## Conclusion

The aged care industry is facing significant challenges with difficulties in staff retention, recruitment, and most importantly, in the quality of patient care. The current study highlighted the need for paying attention to non-clinical skills such as EI (in addition to clinical) for quality care improvement in the aged care industry as well as improving psychological empowerment and wellbeing. The findings suggest that EI contributes to employee empowerment and quality of patient care and adds valuable skills that are important in working with aged care residents and other stakeholders.

## Data Availability

The datasets used and/or analysed during the current study are available from the corresponding author on reasonable request.

## Abbreviations

EI: Emotional intelligence
RMSEA: The Root Mean Square Error of Approximation
CFI: Comparative fit index
GWBQ: General Well-being Questionnaire
EQ-i 2.0®): Emotional quotient inventory
